# The Medical Training of Missionaries

**Published:** 1912-01-20

**Authors:** Edwin A. Neatby

**Affiliations:** Hon. Secretary London Missionary School of Medicine, London Homœpathic Hospital, Great Ormond Street, W.


### The Editor's Letter Box.

### THE MEDICAL TRAINING OF MISSIONARIES.

*To the Editor of* The Hospital.

Sir,—In your issue of December 16 you deplore the fact that missionary societies have been slow to recognise the importance of giving to non-medical foreign missionaries some knowledge of medicine and surgery. You mention the help that has been given in this direction by Livingstone College. You may perhaps be interested to know that work of the same kind has been carried on for the last eight years by the London Missionary School of Medicine. This school has its headquarters in the Homœopathic Hospital, Great Ormond Street, London, W.C., and gives a thorough training in elementary medicine and surgery. Practical work in the wards and out-patient department of the hospital is arranged to suit the needs of the students and the time at their disposal. The school is non-sectarian from a medical as well as from a religious point of view, and a large number of the students are women.—Yours faithfully,

Edwin A. Neatby, M.D., Hon. Secretary

January 13. London Missionary School of Medicine,

London Homœpathic Hospital,

Great Ormond Street, W.

### HOSPITALS UNDER THE NATIONAL INSURANCE ACT.

*To the Editor of* The Hospital.

